# Supplementary material for: Association Between Dietary Intake and Blood Concentrations of One-Carbon-Metabolism-Related Nutrients in European Prospective Investigation into Cancer and Nutrition
Source: Nutrients. 2025 Jun 10;17(12):1970. doi: 10.3390/nu17121970 (PMC12195952; doi:10.3390/nu17121970)

Supplementary Table S1. OCM-related concentration and dietary B-vitamins included in the study, 5th percentile (P5), median (P50), geometric mean (GM), 90th and 99th percentiles.

B-Vitamin	Name	Unit	n	P5	P50	GM	P90	P99
B_2	Riboflavin	nmol/l	10,362	6	15.9	15.9	40.6	153
B_6	Pyridoxal 5′-phosphate (PLP)	nmol/l	10,339	14.1	34.9	34.9	76.9	270.1
B_9	Folate	nmol/l	16,250	5.4	12.9	12.9	28.6	59.3
B_{12}	Cobalamin	pmol/l	16,249	181.5	337.6	337.6	527.2	892.4
-	Methionine	µmol/l	9,015	16.7	25.2	25.2	34.9	49.7
-	Homocysteine	µmol /l	9,353	6.5	10.4	10.4	16	29.5
B_2	Riboflavin	mg/day	16,267	1.2	2.2	2.2	3.2	4.6
B_6	Pyridoxal 5′-phosphate (PLP)	mg/day	16,267	1.1	1.9	1.9	2.9	4.3
B_9	Folate	µg/day	16,267	205.5	369.3	369.3	569.4	846
B_{12}	Cobalamin	µg/day	16,267	2.7	6.1	6.1	11.2	20.5
-	Methionine	g/day	16,267	0.9	1.6	1.6	2.4	3.4
-	Betaine	mg/day	16,267	15.1	113.1	113.1	262.9	450
-	Choline	mg/day	16,267	179.9	313.5	313.5	475.8	696.4
-	Cysteine	g/day	16,267	0.5	0.8	0.8	1.2	1.7

Supplementary Table S2. Information on the type of the blood samples by cancer site-specific study: plasma, serum or a mixture of the two.

**Study**	**Plasma**	**Serum**	**Mix**	**Total**
Breast	4,928	-	-	4,928
Colorectal	3,016	-	-	3,016
Kidney	1,055	-	-	1,055
Lung	-	-	2,209	2,209
Pancreatic	821	-	-	821
Prostate I	-	911	-	911
Prostate II	-	997	-	997
Stomach	797	-	-	797
Upper aero-digestive tract	1,533	-	-	1,533
Total	12,150	1908	2,209	16,267

Supplementary Table S3. Frequency of missing values in blood concentrations and dietary intakes of OCM-related nutrients.

**OCM-Related Nutrient**		**Frequency**
Blood folate	B9	17 (0.1%)
Blood cobalamin	B12	18 (0.1%)
Blood riboflavin	B2	5,905 (36.3%)
Blood PLP[1]	B6	5,928 (36.5%)
Blood methionine	-	7,252 (44.6%)
Blood homocysteine	-	6,914 (42.5%)
Dietary folate	B9	214 (1.3%)
Dietary cobalamin	B12	214 (1.3%)
Dietary riboflavin	B2	214 (1.3%)
Dietary vitamin B6	B6	214 (1.3%)
Dietary methionine	-	214 (1.3%)
Dietary cysteine	-	214 (1.3%)
Dietary betaine	-	214 (1.3%)
Dietary choline	-	214 (1.3%)

[1] PLP: Pyridoxal 5′-phosphate.

Supplementary Figure S1. Heatmap of OCM-related blood concentrations and dietary intakes by vitamin supplement use.

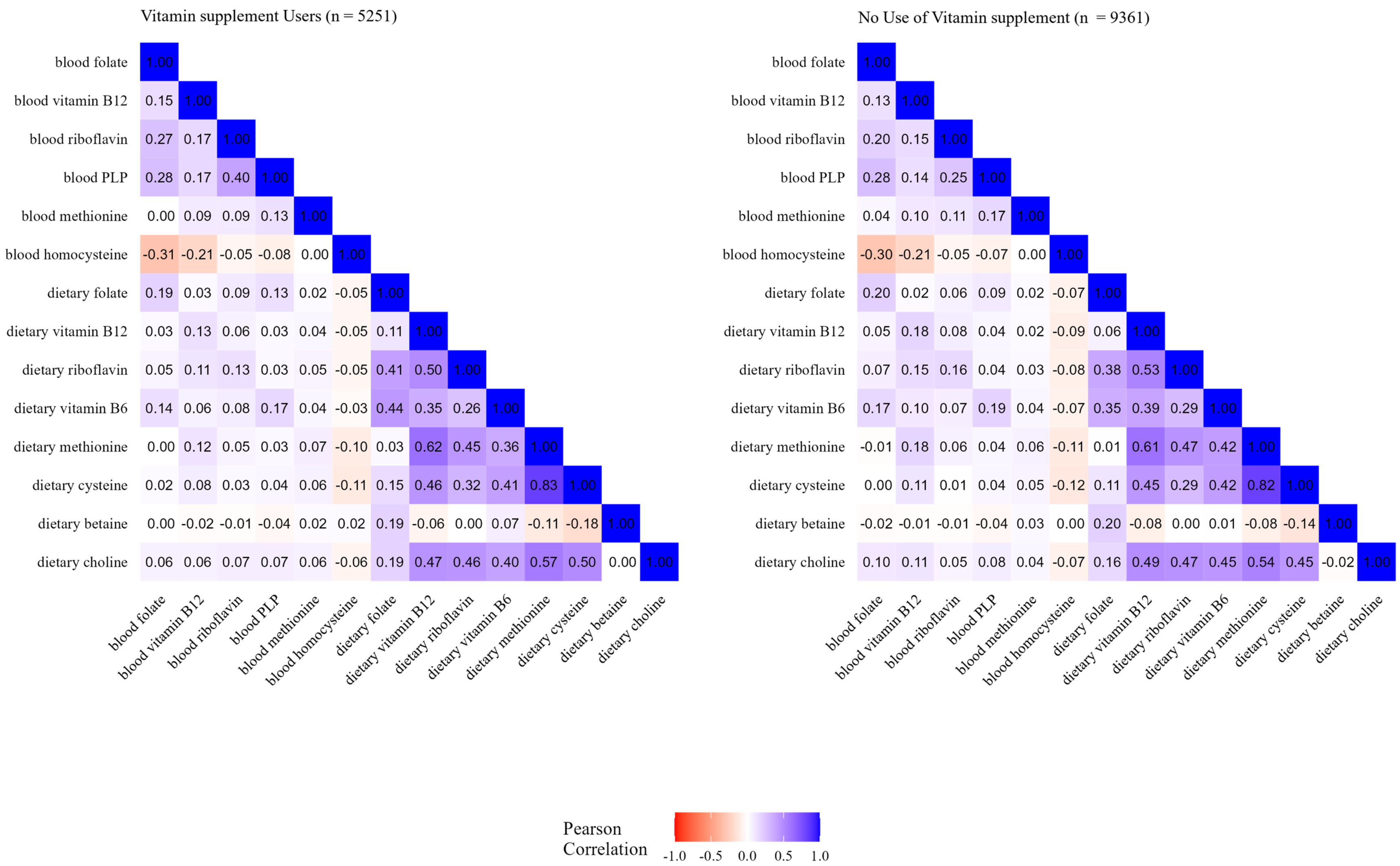

Supplementary Figure S2. Heatmap of OCM-related blood concentrations and dietary intakes by geographical region.

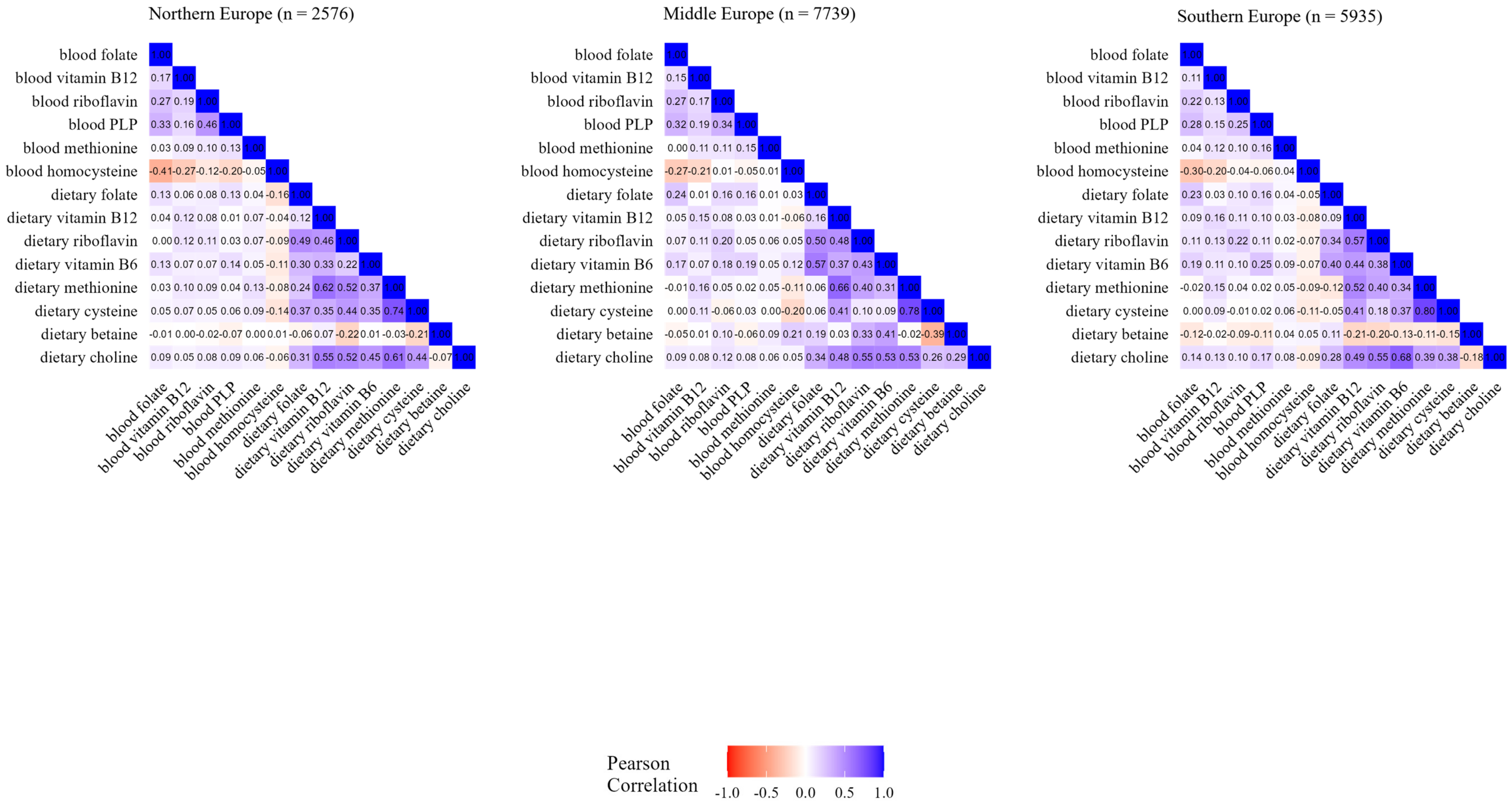

Supplementary Figure S3. Heatmap of OCM-related blood concentrations and dietary intakes by case-control status.

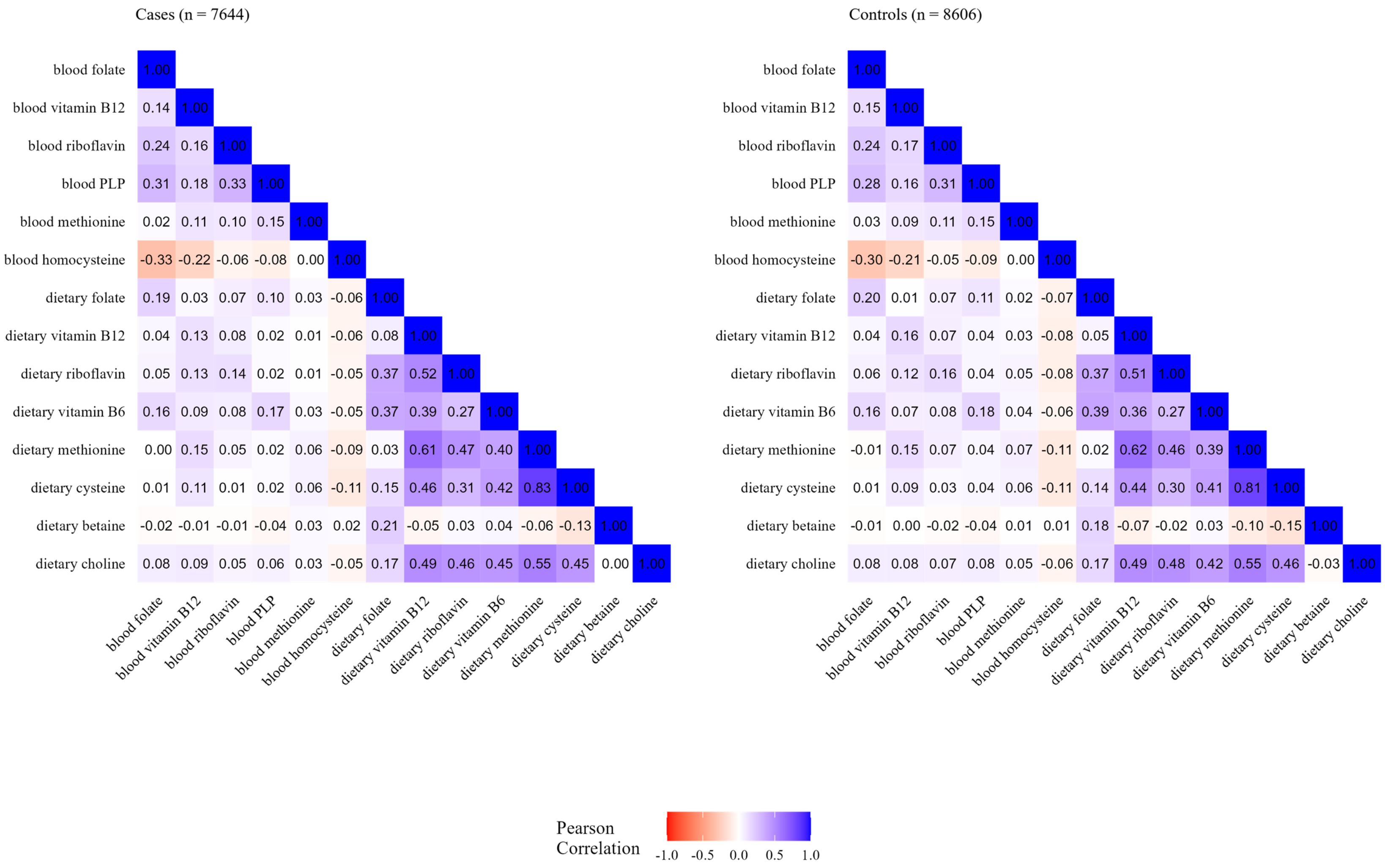

Supplementary Figure S4. Heatmap of OCM-related blood concentrations and dietary intakes by physical activity.

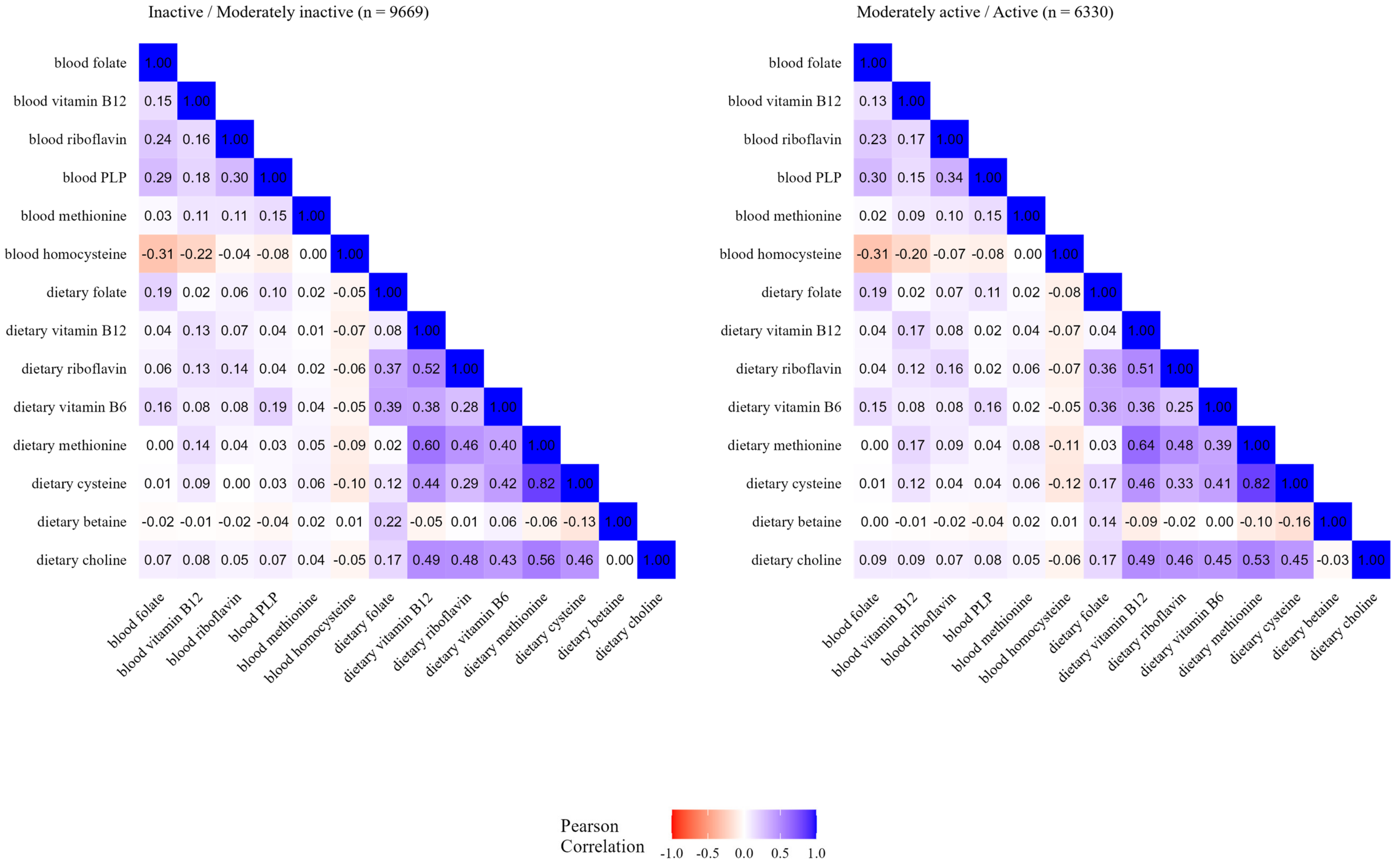

Supplementary Figure S5. Heatmap of OCM-related blood concentrations and dietary intakes by education level.

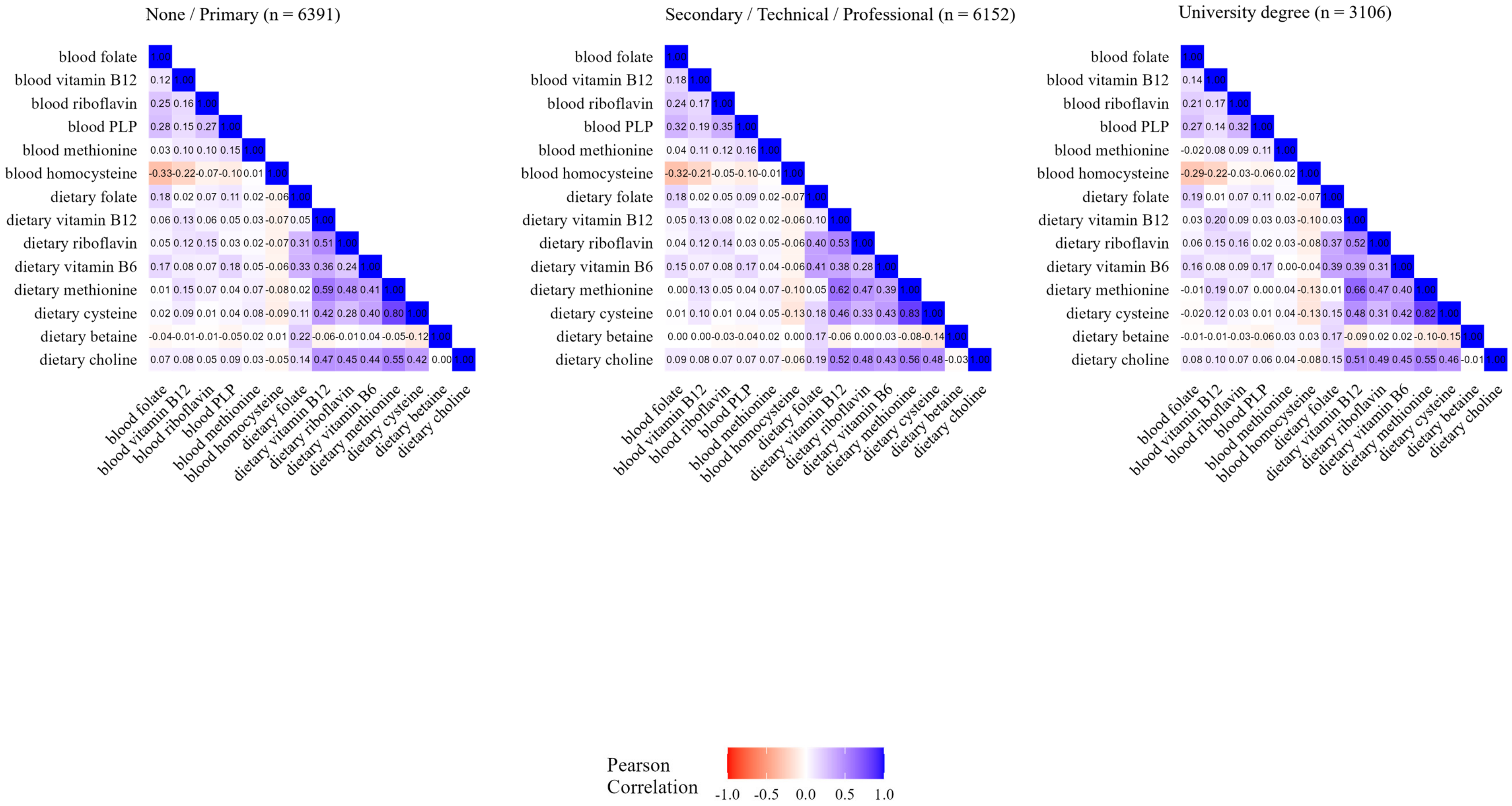

Supplementary Figure S6. Heatmap of OCM-related blood concentrations and dietary intakes – comparison between total dietary folate (left) and dietary folate equivalent, DFE (right).

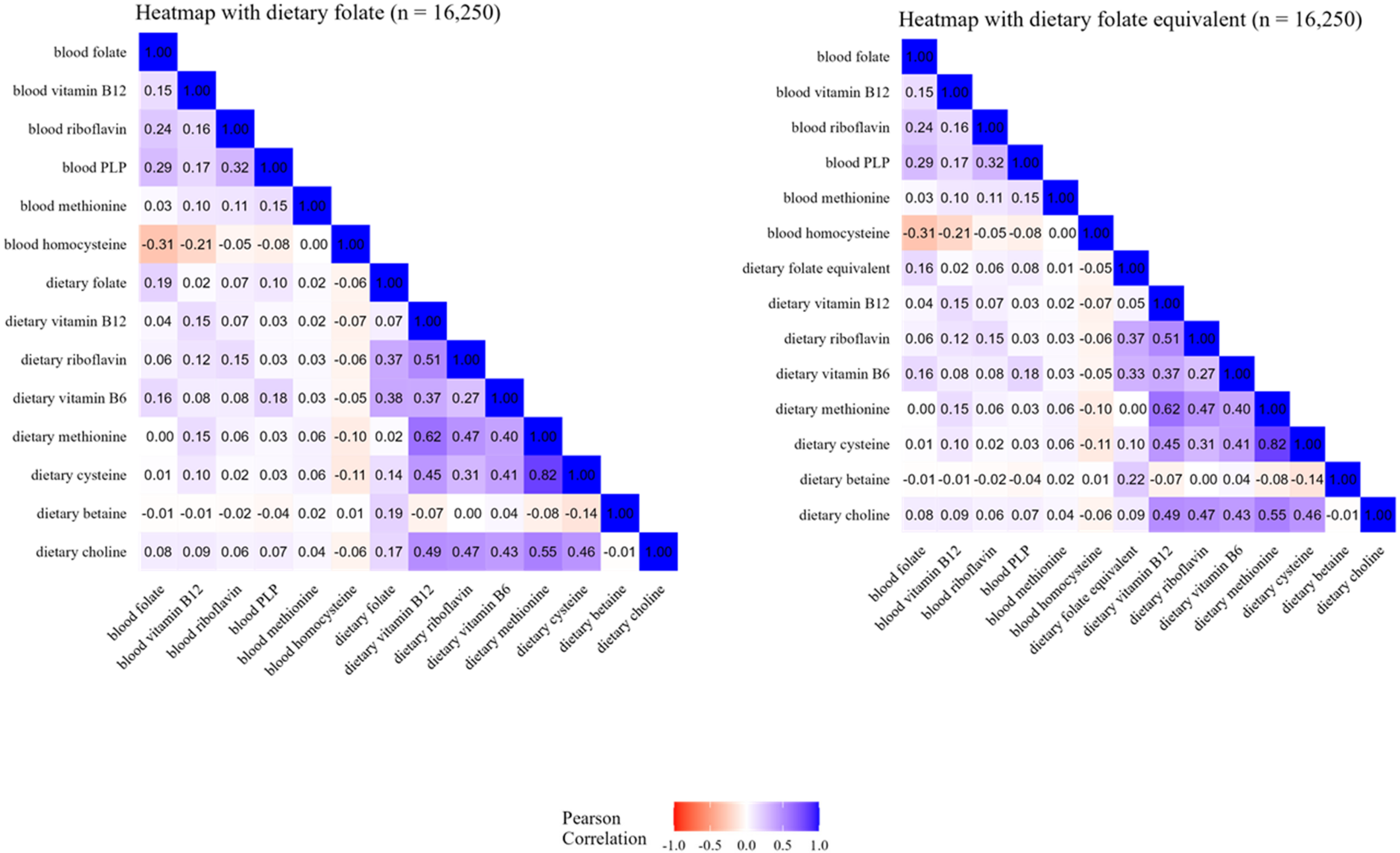

Supplement: Supplementary file 1 [file nutrients-17-01970-s001.zip › nutrients-3608996-supplementary.pdf]
